# HTLV-1/2 is rare among Eastern-European intravenous drug users (IDUs)

**DOI:** 10.1186/1742-4690-12-S1-P79

**Published:** 2015-08-28

**Authors:** Ene-Ly Jõgeda, Radko Avi, Merit Pauskar, Eveli Kallas, Tõnis Karki, Don Des Jarlais, Anneli Uusküla, Irja Lutsar, Kristi Huik

**Affiliations:** 1 Department of Microbiology, University of Tartu, Tartu, Estonia; 2 Department of Public Health, University of Tartu, Tartu, Estonia; 3 Beth Israel Medical Centre, New York, USA

## Background

HTLV-1/2 and HIV likely share the same transmission routes and thus in Europe HTLV occurs mainly among IDUs, interests in clinical implications of HTLV-1/2 co-infection on HIV disease progression have risen. So far the available evidence suggests a protective role of HTLV-2 and adverse effect of HTLV-1 on HIV infection. In Europe, the overall prevalence of HTLV infection is up to 18% among IDUs and less than 1% among blood donors. However, there is no data regarding the overall prevalence of HTLV-1/2 in Eastern-European IDU population where the HIV epidemic mainly lies. We aimed to determine the prevalence of HTLV among IDUs in Estonia and compare it with the rate in healthy volunteers.

## Methods

The study included 345 Caucasian IDUs and 145 healthy volunteers from Estonia. The presence of HTLV-1/2 was determined by nested PCR in 5’ long-terminal repeat region; positive controls were used in every PCR run.

## Results

The analysed IDUs resembled IDUs of HIV epidemic in Estonia: the IDU population was mainly male (79%) with median age of 30 years (interquartile range [IQR] 25-34), and had prolonged duration of illegal drug usage (11 years; IQR=7-14). The rate of co-infections was high – 50% were HIV+, 88% hepatitis C positive, 67% hepatitis B positive. 64% of IDUs reported receptive needle sharing in the past and 18% at least once a month during last six months. None of the IDUs carried HTLV-1 but there was a case of HTLV-2 (prevalence 0.3 %; 95% CI 0.05-0.0162). Healthy volunteers were HTLV-1 and HTLV-2 PCR negative.

## Conclusion

This is the first study investigating the prevalence of HTLV-1/2 among high risk population and healthy volunteers in Estonia. Our results suggest that HTLV is a rare co-infection among IDUs in Estonia.

